# Anxiety and depression and their interdependent influencing factors among medical students in Inner Mongolia: the cross-sectional survey

**DOI:** 10.1186/s12909-022-03839-0

**Published:** 2022-11-14

**Authors:** Qiuxiang Wang, Beisiqi Zhang, Song Zhang, Chengxi Wei, Danni Fu, Honglin Zhao, Xue Bai

**Affiliations:** 1 Inner Mongolia Key Laboratory of Mongolian Medicine Pharmacology for Cardio-Cerebral Vascular System, Inner Mongolia Minzu University, No. 996, Xilamulun Street (West), Horqin District, 028000 Tongliao, China; 2 Department of Preventive Medicine, Medical College, Inner Mongolia Minzu University, No. 996, Xilamulun Street (West), Horqin District, 028000 Tongliao, China; 3 Disease Control And Prevention, Health commission, No. 11 Jianguo Road, Horqin District, 028005 Tongliao, China

**Keywords:** Anxiety, Depression, Medical students, Diet-related factors, Inner Mongolia

## Abstract

**Background:**

Mental health has become a global problem, among which anxiety and depression disorder were ranked as the first and sixth leading causes of disability, respectively, according to the World Health Organization (WHO). Medical students experienced higher levels of anxiety and depression than the general population. But there was a lack of research on the emotional situation among medical students in Inner Mongolia. The main objectives of this study were to investigate the prevalence of anxiety and depression symptoms as well as the factors that influence them among medical students in Inner Mongolia.

**Methods:**

A cross-sectional study was conducted on 1282 students from a university in Inner Mongolia, China, ranging in age from 16 to 27 years. They were assessed demographic indicators, the disorder of anxiety and depression using Zung’s Self-Rating Anxiety Scale and Self-Rating Depression Scale (SAS and SDS) by an anonymous, self-administered questionnaire. The internal reliability and validity of the questionnaire were determined using Cronbach’s alpha coefficient, Kaiser-Meyer-Olkin (KMO), and Bartlett’s sphericity. T-tests and one-way ANOVA were used to explore factors, including demographic and behavioral information influencing anxiety and depression disorder. According to the above results of exploring the influencing factors based on univariate analysis, significant factors (p < 0.05) were entered into multiple linear regressions that sequentially fitted to predictors associated with anxiety and depression. The collected data were entered into EpiData for windows and analyzed using SPSS 26.0. The *p* < 0.05 was considered to be significantly different.

**Results:**

The questionnaire was completed by 1187 students with a 92.59% response rate. The prevalence of anxiety and depression symptoms among medical students were 10.36% and 24.43%, and the mean ± standard deviation (M ± SD) anxiety and depression scores were 39.60 ± 7.81 and 48.23 ± 9.06, respectively, among the medical students. The specific contributions of the two scales with good reliability and validity were 60.58% and 63.59%, respectively. For univariate analysis, age, whether the daily meal was at a fixed time, grade, the birthplace of students, average daily eating habits, were the factors that influenced both the total score of SAS and SDS (p < 0.05). For further analysis, the results showed that “Birthplace of students” and “Whether daily meals at a fixed time” were significantly associated with anxiety and depression. Furthermore, “Age” and “Mode of delivery” were independent risk factors for depressive disorder.

**Conclusion:**

Our findings revealed that high prevalence of mental health problems among medical students in Inner Mongolia. The Ministry of Medical Education should make a targeted intervention for specific risk factors of this study to improve psychological well-being and face uncertain future challenges among university students in Inner Mongolia.

**Supplementary Information:**

The online version contains supplementary material available at 10.1186/s12909-022-03839-0.

## Background

Global Burden of Diseases (GBD) 2019 showed that mental disorders have reached the top ten leading causes of burden worldwide since 1990 [1]. Gradually mental disorders have been increasingly recognized as leading causes of disease burden, even emerged as one of the leading causes of morbidity in the population [2]. Every year, more than 10 million people attempt suicide around the world, with half of them suffering from psychological issues and mental disorders such as anxiety disorders, mania, and nonaffective psychosis [3]. Apathy, irritability, and major depressive or psychotic symptoms are examples of mental disorders that can disrupt people’s normal lives and social development. Anxiety and depression were considered the most common mental disorders, accounting for 13.60–28.80%, respectively, in the general population [4]. Anxiety was characterized by a sense of tension, nervousness, worried thoughts, and physical changes such as sweating, trembling, and increasing blood pressure [5]. Depression is a mental disorder characterized by depressed mood, loss of pleasure, reduced energy and activity, decreased self-esteem, decreased attention with changes in appetite, and sleep disturbances, and it can lead to suicidal ideation and behavior [6].

As a particular population endured a critical transition from adolescence to adulthood at when both brain and cognition undergo fundamental developmental change and reorganization. The critical transition with experiencing physical growth and endocrine disorders was particularly vulnerable period, therefore university students might make significant decisions and face challenges [7]. Previous studies reported that anxious and depressive symptoms were increasingly noted among university students worldwide [6]. Especially medical students might experience a higher rate of anxiety and depression, which were as high as 28.00% and 33.80% compared to other specialties of university students [8–10].

As we all know, medicine is one of the most competitive fields with a combination of theory and an intense course load during education, which might more probably lead to the emergence of negative emotions [11]. Furthermore, the high workload caused by an imbalanced doctor-patient ratio in China’s medical system may cause medical students to experience more psychological problems than in Western or other Asian countries [8]. A growing body of previous evidences also suggested that long work hours adversely affected mental health across a variety of domains [12]. In addition, sleep quality as a major factor affected the mood of medical students by disturbing students’ learning abilities, academic performance, and interpersonal relationships [13]. With the Coronavirus disease 2019 (COVID-19) spreading rapidly and widely worldwide, more than 30 thousand people were confirmed as infected by June 2020 [14]. During the epidemic of COVID-19, medical staff might face challenges in coping with unknown emergencies and high-risk clinical environments, which might further impact their professional identity and lead to negative emotional symptoms for medical students [15, 16].

As the northwest frontier of China, the education and other resources in Inner Mongolia lagged behind the area of economic development. Due to the remote geographical location of Inner Mongolia, medical education was also relatively backward. Therefore, our study aimed to evaluate the anxiety and depression levels by SAS and SDS (Zung’s Self-Rating Anxiety Scale and Self-Rating Depression Scale) and to explore the influencing factors, including conventional demographic and special behavior information according to the actual situation among all the medical students in Inner Mongolia Minzu University [14]. It is hoped that the related education department will take targeted measurements to improve mental health according to our future research results for Inner Mongolia medical students.

## Methods

### Study design

This cross-sectional study was conducted from November to December 2021.

### Participants

The searched sampling frame consisted of all medical students from the Medical College of Inner Mongolia Minzu University. The research chosen respondents who were eligible to participate in the survey during whole process by the cluster sampling method. The cluster sample method was grouped all the samples into different classes, which is called group, and then the group was used as a sampling unit to sample individual information by simple random sampling. The sampling procedures were probabilistic and conducted without replacement at all stages.

### Settings

Participants were recruited all medical students from a public school with medical specialty in Inner Mongolia, including five medical specialties: Preventive medicine (Five-year), Medical imaging technology (Four-year), Medical laboratory technology (Four-year), Pharmaceutical preparation (Four-year), and Medical laboratory technology ISEC (Four-year).

### Sampling procedure

All medical students should participate in the study expect for students who are unwilling or unable to complete the whole investigation due to personal reasons. The trained researchers explained the purpose and content of the study to the students before emphasizing the principle of voluntarily, anonymity, and confidentiality prior to investigations. The informed consent form was then distributed to the medical students with the age of more than 18 years old. On the other hand, those researched students with the age of below 18 had obtained the informed consent from the guardians before survey by the form of message or email. And all the entire process took about 30 min. After the students completed the questionnaire, the researchers tracked the number of questionnaires and checked the accuracy and completion situation in the background of the network. As problems arose during the process, researchers would communicate with student leaders. IP address restriction technology was used to ensure that students with the same IP address completed the questionnaires only once. All information was collected simultaneously, and incorrect or missing data were not included in the analysis.

### Questionnaire

The questionnaire in the present study included three parts.

The first part was designed to collect demographic information such as name, specialty, grade, gender, age, ethnicity, the birthplace of students, whether one child in the family, family status (parents’ marital status), mode of delivery, birth weight, feeding way at birth, family history of cardiovascular diseases, family history of diabetes, the daily motor activity patterns and dining habits.

In the second part, we used the self-rating Anxiety Scale (SAS), a widely used symptom measurement tool that can evaluate the level of anxiety symptoms to reflect the subjective feelings of psychiatric helpers. It was compiled by W.K. Zung in 1971 and contains four components with 20 items, including the mixed symptom of anxiety and autonomic nerve function, autonomic nerve dysfunction, exercise tension, and anxious mood. There are 15 positive scores and 5 reverse scores (marked with * in items). The participants scored each item on a 4-point Likert scale based on the frequency of symptoms in the past 7 days: 1-never or very rarely, 2-sometimes, 3-most of the time, and 4-most or all of the time. The total score of the question is obtained from the original score by adding each item and then multiplied by 1.25 to calculate the final standard score. The higher scores mean, the more severe anxiety symptoms. The cut-off value of the SAS standard score is defined as: no anxiety (< 49 points), mild (50–59 points), moderate (60–69 points), and more than 70 points are severe anxiety [17]. The Chinese version of SAS was used in the survey, which had good reliability and validity confirmed in previous studies (see in Supplementary Material) [18].

In the third part, we used the self-rating depression scale (SDS) as a widely used symptom measurement tool that can evaluate the level of depressive symptoms by reflecting the subjective feelings of psychiatric helpers. It was compiled by William W.K. Zung in 1965 [19] and contained four components with 20 items, including mental-emotional symptoms, physical, psychomotor disorders, and psychological disorders. There were 10 positive scores and 10 reverse scores (with *). The participants scored each item on a 4-point Likert scale based on the frequency of symptoms in the past 7 days: 1-a little of time, 2-some of time, 3-good part of the time, and 4-most of the time. The total score of the question is obtained from the original score by adding each item and then multiplied by 1.25 to calculate the final standard score. The higher scores mean more serious depression symptoms. The cut-off value of the SDS score is defined as follows points: no depression (< 49), mild (50–59), moderate (60–69), and severe (> 70) [17]. The Chinese version of SDS was used in the survey with good reliability, and its validity has been confirmed in previous studies (see in Supplementary Material) [18].

### Statistical analysis

The collected data was input into EpiData for windows and analyzed by SPSS 26.0. The continuous variables results were expressed by mean ± standard deviation (M ± SD) or median and interquartile range (IQR). In general, our sample was large, which can be considered a normal distribution according to the central limit theorem. The Cronbach’s alpha coefficient was used to determine the instrument’s internal consistency. Kaiser-Meyer-Olkin (KMO) and Bartlett’s sphericity test were used to check the suitability of the exploratory factor analysis. Independent sample *t*-test and one-way ANOVA tests were used to identify the influencing factors of anxiety and depression. According to the above results, multiple linear regressions would further explore the independent factors. Moreover, a univariate general linear model was used to find the interaction effects among independent influencing factors. The *p*-value < 0.05 was considered to be significantly different.

## Results

### Sociodemographic characteristics of participants

A total of 1282 questionnaires were distributed to the medical students at Inner Mongolia Minzu University. Following the investigation, a total of 1187 questionnaires completed qualified to be analyzed, with a response rate was 92.59%. The age of participants ranged from 16 to 27 years, with an average of 20.13 ± 1.62 years. The number of students from grade 1 to grade 5 was 324 (27.30%), 340 (28.64%), 265 (22.33%), 213 (17.94%) and 45 (3.79%) respectively. Among the participants were 413 male students (34.79%) and 774 female students (65.21%). Table 1 presents the detailed demographic characteristics of the participants.

**Table 1:** Demographic characteristics of the study population (*N* = 1187)

Variables	N(%)
Specialty	
Preventive medicine Medical imaging technology Medical laboratory technology Pharmaceutical preparation Medical laboratory technology ISEC	279(23.50%) 413(34.79%) 279(23.50%) 74(6.23%) 142(11.96%)
Grade	
1 2 3 4 5	324(27.30%) 340(28.64%) 265(22.33%) 213(17.94%) 45(3.79%)
Gender	
Male Female	413(34.79%) 774(65.21%)
Age	
16–18 19 20 21 22–27	142(11.96%) 293(24.68%) 232(19.55%) 240(20.22%) 280(23.59%)
The birthplace of students	
Urban Rural	454(38.25%) 733(61.75%)
One child in the family	
Yes No	506(42.63%) 681(57.37%)
Family status	
Good marital status Divorced parent Single parent family Other	1035(87.19%) 77(6.49%) 39(3.29%) 36(3.03%)
Mode of delivery	
Natural delivery Cesarean section Both Unclear	756(63.69%) 202(17.02%) 63(5.31%) 166(13.98%)
Family history of diabetes	
Father Mother Both Neither	40(3.37%) 17(1.43%) 2(0.17%) 1128(95.03%)
The average numbers of daily meals	
< 3 times 3 times > 3 times	291(24.52%) 863(72.70%) 33(2.78%)
Whether daily meals at a fixed time	
Yes Occasionally Not occasionally No specific rules	480(40.44%) 207(17.44%) 393(33.11%) 107(9.01%)
Daily eating habits	
Primary on cereals-based diet Primary on meat-based diet Primary on vegetables-based diet Balanced collocation No specific rules	190(16.01%) 95(8.00%) 71(5.98%) 570(48.02%) 261(21.99%)
The daily motor activity patterns	
Primary on outdoor activity Primary on indoor activity Both No specific rules	181(15.25%) 102(8.59%) 286(24.09%) 618(52.06%)

### Reliability and validity

We used Cronbach’s α to confirm the reliability of the scales and a principal component analysis with a varimax rotation to calculate the Bartlett test of sphericity, Kaiser-Meyer-Olkin (KMO) for evaluating the suitability of exploring factor analysis. As shown in Table 2, reliability was satisfactory (Cronbach’s α > 0.75) for the two scales. Preliminary tests for exploratory factor analysis showed acceptable values with Kaiser-Meyer-Olkin (KMO = 0.86, 0.90) and Bartlett’s test of sphericity (χ2 = 6488.06, 8061.21) p < 0.01, indicating that the correlations were sufficient for analysis. Table 2 presents the results of our study in detail with reliability and validity.

**Table 2:** The reliability and validity of SAS and SDS

Scale	Cronbach α	KMO	Bartlett’s Sphericity		Cumulative contribute rate
			Test Ki-Kare Value (χ²)	Significance level (sig.)	
SAS	0.75	0.861	6488.06	*P* < 0.01	60.58%
SDS	0.78	0.898	8061.21	*P* < 0.01	63.59%

### The symptom of anxiety and depression among medical students

**Fig. 1:**
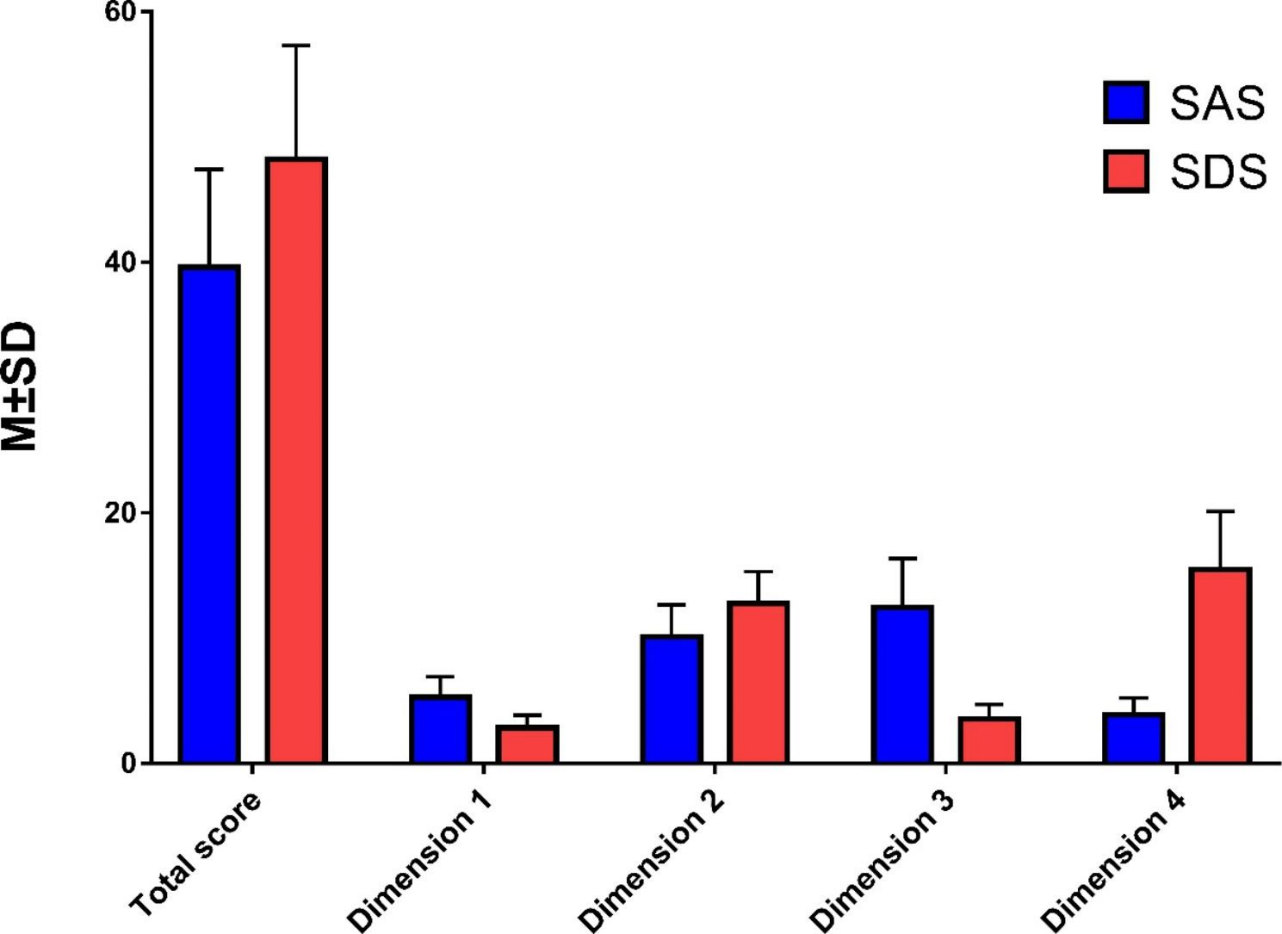
Descriptive analysis of the total score and each dimension of SAS and SDS

The SAS score of participants ranged from 25 to 76, with an average score of 39.60 ± 7.81, in which 123 (10.36%) students reported anxiety symptoms. The SDS score ranged from 25 to 76 with an average score of 48.23 ± 9.06, and 290 (24.43%) participants showed depression symptoms. The detailed information is shown in Table 3; Fig. 1.

**Table 3:** Descriptive analysis of the total score and each dimension of SAS and SDS

Categories	Min ~ Max	M ± SD
SAS (Total score)	25 ~ 76	39.60 ± 7.81
Dimension 1:Anxious mood	4 ~ 15	5.28 ± 1.64
Dimension 2:Autonomic nerve dysfunction	8 ~ 26	10.09 ± 2.57
Dimension 3: Exercise tension	6 ~ 22	12.43 ± 3.91
Dimension 4:The mixed symptom of anxiety and autonomic nerve function	2 ~ 8	3.88 ± 1.35
SDS (Total score)	26 ~ 75	48.23 ± 9.06
Dimension 1: Mental-emotional symptoms	2 ~ 8	2.84 ± 1.01
Dimension 2: Physical	8 ~ 23	12.76 ± 2.54
Dimensions 3: Psychomotor disorders	2 ~ 7	3.58 ± 1.13
Dimensions 4: Psychological disorders	8 ~ 29	15.45 ± 4.66

M ± SD: mean ± standard deviation

### Factors influenced the level of anxiety and depression among medical students

To test demographic characteristics that may influence SAS and SDS scores, t-tests and one-way ANOVA tests were used. Comparisons within the various demographic characteristics revealed that age, whether the daily meal was at a fixed time, grade, the birthplace of students, average daily eating habits, the average number of daily meals, and daily intake of other snacks were the factors that influenced the total score of SAS (*p* < 0.05). Age, whether daily meals were at a fixed time, grade, the birthplace of students, daily eating habits, and mode of delivery were the primary influencing factors on the total score of SDS (*p* < 0.05). (Table 4)

**Table 4:** Univariate analysis to identify influencing factors associated with anxiety and depression symptoms

	SAS					SDS				
Variables	Total scores	Dimension 1	Dimension 2	Dimension 3	Dimension 4	Total scores	Dimension 1	Dimension 2	Dimension 3	Dimension 4
Age										
16–18	38.81	5.43	10.13	11.75	3.74	42.05	3.01	12.45	3.37	14.72
19	39.79	5.49	10.4	12.01	3.93	43.87	3.05	13.12	3.6	15.24
20	38.99	5.23	10.05	12.22	3.69	42.32	2.76	12.52	3.47	14.99
21	39.09	5.02	9.68	12.68	3.89	42.76	2.65	12.68	3.6	15.17
22–27	40.74	5.23	10.13	13.17	4.06	45.05	2.78	12.79	3.74	16.65
F	2.53^*^	4.06^**^	3.18^*^	4.92^**^	2.86^*^	4.27^**^	7.30^**^	2.54^*^	3.07^*^	6.58^**^
Grade										
1	38.5	5.27	10.03	11.71	3.79	41.95	2.94	12.52	3.43	14.58
2	40.89	5.54	10.43	12.79	3.96	45.23	2.99	13.28	3.78	16.04
3	38.19	5.01	9.77	11.95	3.82	40.81	2.6	12.27	3.35	14.32
4	40.7	5.08	9.92	13.51	4.04	45.93	2.73	12.94	3.81	17.18
5	40.81	5.84	10.58	12.56	3.67	43.40	3.04	12.53	3.42	15.60
F	7.52^**^	5.36^**^	2.92^*^	8.39^**^	1.82	15.35^**^	8.35^**^	7.34^**^	9.17^**^	14.77^**^
The birthplace of students										
Urban	39.00	5.32	10.23	11.93	3.72	42.61	2.96	12.69	3.42	14.92
Rural	39.97	5.25	10.00	12.73	3.99	43.89	2.77	12.8	3.68	15.77
T	-2.07^*^	0.71	1.51	-3.54^**^	-3.39^**^	-2.42^*^	3.19^**^	-0.78	-3.78^**^	-3.05^**^
Gender										
Male	39.75	5.14	9.76	13.00	3.90	43.81	2.68	12.66	3.67	15.95
Female	39.52	5.35	10.27	12.12	3.87	43.40	2.93	12.81	3.53	15.18
T	0.49	-2.16^*^	-3.29^**^	3.49^**^	0.36	1.14	-4.05^**^	-1.01	1.97*	2.59^*^
Specialty										
Preventive medicine	38.87	5.51	10.24	11.66	3.68	42.98	2.95	12.64	3.43	15.27
Medical imaging technology	40.07	5.12	9.89	12.97	4.07	43.99	2.77	12.88	3.69	15.77
Medical laboratory technology	39.34	4.99	9.76	12.78	3.94	43.37	2.68	12.6	3.63	15.68
Pharmaceutical preparation	41.35	5.86	11.08	12.31	3.82	43.45	3.05	13.04	3.49	15.05
Medical laboratory technology ISEC	39.25	5.51	10.51	11.73	3.65	42.58	3.08	12.80	3.50	14.60
F	2.07	7.02^**^	4.78^**^	6.96^**^	5.19^**^	0.91	6.12^**^	0.91	2.87^*^	2.42^*^
Whether one child in the family										
Yes	39.89	5.24	10.16	12.60	3.91	42.99	2.82	12.65	3.52	15.29
No	39.38	5.30	10.04	12.30	3.86	43.72	2.86	12.84	3.62	15.56
T	1.13	-0.64	0.83	1.33	0.56	-1.37	-0.59	-1.26	-1.47	-0.96
Whether daily meals at a fixed time										
Yes	39.01	5.07	9.85	12.39	3.89	42.36	2.73	12.59	3.51	14.96
Occasionally	40.98	5.48	10.30	13.09	3.92	44.85	2.92	13.23	3.76	15.88
Not occasionally	38.79	5.25	10.12	11.88	3.78	42.98	2.87	12.57	3.51	15.33
No specific rules	42.54	5.88	10.66	13.34	4.15	46.84	3.10	13.29	3.79	17.20
F	9.75^**^	7.43^**^	3.67^*^	7.08^**^	2.27	9.53^**^	4.90^**^	5.45^**^	4.10^**^	7.60^**^
Daily intake of other snacks										
Never	43.59	4.50	10.38	15.25	4.75	49.25	3.13	12.38	4.38	19.50
There is very little	40.50	5.15	10.07	13.14	4.05	43.94	2.65	12.86	3.58	15.96
Once in a while	38.93	5.13	9.84	12.35	3.83	43.00	2.77	12.65	3.59	15.31
Often	40.09	5.63	10.54	12.02	3.87	43.39	3.02	12.92	3.53	15.13
Almost every day	42.73	5.92	11.15	13.07	4.05	46.13	3.32	13.22	3.60	16.63
F	3.93^**^	6.11^**^	5.00^**^	2.44	1.88	1.77	6.18^**^	0.95	1.10	2.34
Daily eating habits										
Primary on cereals-based diet	39.86	5.28	9.94	12.63	4.04	44.06	2.84	12.88	3.65	15.78
Primary on meat-based diet	40.54	5.81	10.46	12.28	3.87	43.42	3.09	12.42	3.64	15.47
Primary on vegetables-based diet	39.6	5.54	10.55	11.8	3.79	43.58	3.00	12.85	3.54	15.42
Balanced collocation	38.82	5.06	9.86	12.34	3.79	42.39	2.75	12.67	3.48	14.92
No specific rules	40.77	5.49	10.43	12.7	4.00	45.10	2.92	12.97	3.74	16.34
F	3.31^*^	5.74^**^	3.18^*^	0.99	1.85	4.39^**^	3.58^**^	1.18	2.71^*^	4.50^**^
Mode of delivery										
Natural delivery	39.48	5.30	10.12	12.29	3.86	43.33	2.85	12.76	3.57	15.39
Cesarean section	39.11	5.29	10.09	12.11	3.79	41.75	2.85	12.5	3.37	14.6
Both	38.85	5.25	10.32	11.71	3.79	42.10	2.9	12.48	3.57	14.6
Unclear	41.02	5.14	9.85	13.69	4.13	46.26	2.79	13.2	3.89	17.05
F	2.35	0.47	0.69	7.01^**^	2.40	8.32^**^	0.25	2.66^*^	6.61^**^	9.30^**^
The daily motor activity patterns										
Primary on outdoor activity	38.31	5.10	9.71	11.99	3.85	42.60	2.76	12.71	3.48	15.03
Primary on indoor activity	39.4	5.39	9.96	12.46	3.71	42.17	2.73	12.77	3.54	14.58
Both	39.84	5.06	9.69	13.19	3.93	43.27	2.64	12.83	3.66	15.4
No specific rules	39.89	5.41	10.41	12.20	3.90	43.90	2.98	12.74	3.58	15.73
F	2.04	4.08^**^	7.02^**^	4.15^**^	0.79	1.76	8.55^**^	0.11	1.05	2.80^*^
The average number of daily meals										
< 3 times	40.54	5.54	10.44	12.59	3.87	44.40	2.96	13.14	3.57	15.76
3 times	39.19	5.16	9.95	12.36	3.88	43.02	2.79	12.62	3.58	15.33
> 3 times	41.86	5.97	10.55	12.91	4.06	44.64	3.24	12.85	3.76	15.70
F	4.10^*^	7.17^**^	4.44^*^	0.64	0.31	2.84	5.88^**^	4.61^*^	0.42	0.97

^*^p < 0.05; ^**^p < 0.01

### Multiple linear regression

After univariate analysis, multiple linear regressions were sequentially fitted to predictors and screened the risk factors associated with anxiety and depression further. It was in order to identify partial contribution of each predictor to overall multiple regression calculation based on above given significant factors, and it was reported by the standardized and non-standardized regression coefficients. In our study, multiple linear regression (MLR) analysis was performed using influencing factors based on the results of the univariate analysis as independent variables. SAS and SDS total scores were applied as the dependent variable, only two independent variables were retained in SAS total score, and four independent variables were indicated in SDS total score (*P* < 0.05). It can be concluded that “Birthplace of students” and “Whether daily meals at a fixed time” were the essential independent influencing factors and effective predictors of SAS total score (*P* < 0.05). “Age, mode of delivery, the birthplace of students, and whether daily meals at a fixed time” were the critical influencing factors and effective predictors of SDS total score (*P* < 0.05). Table 5 depicts the results of the MLR of anxiety and depression symptoms.

Furthermore, the General Linear Model with interaction analysis was used, and the results showed no interaction differences influencing the total SAS and SDS scores (*P* > 0.05).

**Table 5:** Results of independent influencing factors of SAS/SDS in the multiple linear regression model

SAS					
Variable	B	SE	β	t	*P*
The birthplace of students	0.91	0.47	0.06	1.95	0.05
Whether daily meals at a fixed time	0.44	0.22	0.06	2.02	0.04
SDS					
The birthplace of students	1.02	0.54	0.06	1.90	0.06
Age	0.52	0.19	0.08	2.68	0.01
Whether daily meals at a fixed time	0.86	0.25	0.10	3.42	0.00
Mode of delivery	0.66	0.24	0.08	2.78	0.01

## Discussion

Anxiety and depression among medical students were a common mental disorder and had become an increasingly severe problem recently. Our study aimed to measure the situation and explore influencing factors of anxiety and depression symptoms among medical students at Inner Mongolia Minzu University. The present results demonstrated that the prevalence of anxiety and depression was 10.36% and 24.43%, respectively, similar to the previously reported results from medical students in China and Nigeria [20, 21]. It might mean that depression was a more vulnerable and sensitive symptom than anxiety for medical students in the present survey. Furthermore, we were interested in discovering that “the diet-related factors about whether daily meals at a fixed time” and “birthplace of students” as independent influencing factors were significantly associated with the level of anxiety and depression (p < 0.05).

In this study, “Whether daily meals at a fixed time” was associated with the level of anxiety and depression (p < 0.05). Students with “No specific rules in daily meals time” might lead to obesity due to a change in intestinal flora disorder, which could affect brain function and increase the risk of mental illnesses like anxiety and depression [22]. On another hand, an irregular diet influenced the microbiota-gut-brain axis as a bi-directional communication pathway, contributing to brain function and neurodegenerative diseases [23]. The myriad and complex relationships that can exist between diet and psychiatric symptoms. The over-eating could be caused by stress through the brain to lead to disorders such as depression due to functional and social impairments. Studies have showed the possible mechanism between a link between diet and stress-related psychiatric disorders was formed and strengthened by neurotransmitters and neuropeptides [24]. In fact, medical students usually had a common phenomenon of irregular diet due to learning many professional courses, lectures, basic experiments, clinical practice, and related comprehensive courses compared to other students, as evidenced by some previous studies [25]. Moreover, people living in Inner Mongolia usually consume beef, milk, high-fat, and high salt in their morning diet, which might increase the risk of emotional problems from intestinal flora [26, 27].

In the present study, “The birthplace of students” was significantly associated with the level of anxiety and depression (p < 0.05). Students from rural areas scored higher in anxiety (39.97 ± 7.85) and depression (43.89 ± 9.27) than those from urban areas. Though China had a transition from a dual-structure society to urban-rural development, college students from the urban area will find it easier to adapt to the new environment and better to learn the diversity of knowledge due to their relatively complicated and varied original living environment with rich education and economic resources, diverse life experiences, and so on [28]. Furthermore, many students in rural areas are left-behind children (LBC) with a lack of parental care, guidance, and communication. They faced extraordinary pressure and emotional difficulties in deciding on their future lives [29, 30]. Studies showed that people with relatively lower education levels are more likely to have lower family professional statuses and society support, more difficulty integrating into society, and less access to healthcare which made worse mental states [31]. Inner Mongolia is located in the border area with low and typical tendency in expenditure of educational and medical resources, the resource shared to rural areas were relatively insufficient. Further, medicine is a unique major that needs lifelong learning and practice; the students might perform better with positive growth experiences and accumulated knowledge early on.

“Age” and “Mode of delivery” were significantly associated with the level of depression (p < 0.05) only. The oldest age group consisted of fourth and fifth-grade students who scored highest on anxiety (40.74 ± 7.94) and depression (45.05 ± 9.50). Senior medical students had suffered a common mental illness that may be attributed to the gap between clinical practice reality and ideal in a transition period and foreseeable competition for the unclear further related postgraduate examination and employment pressure [25]. Moreover, the students with “Natural delivery” had a higher score for anxiety (39.48 ± 7.91) and depression (43.33 ± 9.10) in this study. In previous studies, cesarean section was associated with an increased risk of mental symptoms in the offspring. In previous studies, cesarean section was associated with an increased risk of mental symptoms in the offspring, the reason was that women with cesarean section might have higher pain of somatization, depression, and anxiety symptom levels than those who had natural or vaginal delivery [32]. The cesarean section mothers were tend to confide the situation to their children which might affected their emotion [33]. Our conclusion was contrary to the findings of this study, the reason might be that the medical students had in-depth insights into physiology and related medical knowledge. Thus, they might be more rational to face the relationship between psychological and physical performance.

We also found medical students with different some demographic factors such as gender and ‘whether the only child in the family’ had different emotion situation just without statistically significant differences (p > 0.05). Some research pointed out that male students were more likely to contact more seductive news due to the higher usage of mobile cellphones which might cause poor sleep quality and negative symptoms [34]. The only child in the family would more likely to have low emotion independence and high emotional insensitivity caused by being protected and caring with high baby schema from their parents [35]. In addition, they need face and solve any difficult and challenge from family or society only. Therefore, the only child might have lower emotional situation like high level of anxiety or depression which was consist with our results [35]. However, for aspects of statistical differences, we should obtain more sample and more accurately methods to search and analyze them according research actual situation in the further.

### Strengths and Limitations

The study had some strengths. Firstly, our sample size was very large. Although there were potential influencing factors to affect the anxiety and depression symptoms among medical students, few studies had a large enough sample and focused on prevalence rates related to both depression and anxiety symptoms. And then while there was an increasing large of articles on exploring potential risk factors to the prevalence of depression and anxiety symptoms among medical students, about the researchers of association between ‘eating habits’ and ‘mental health’ were lack in Inner Mongolia. However, the findings of this study should consider the following limitations. First, this was a cross-sectional observational study, which could not determine the time course of the causal relationships between the variables. In order to confirm the conclusion, further longitudinal and more diverse investigations are needed. Second, the study population consisted only of Inner Mongolia Minzu University medical students who cannot be represented as generalized medical students in whole Inner Mongolia. Future research should be conducted on more populations. Third, the influencing factors should be restricted by designed questions, and the follow-up study could include more diet and behavior habits factors according to our results.

## Conclusion

The results of the present study suggested a strong relationship between the “Birthplace of students” and the level of anxiety/depression symptoms. Unexpectedly, we also found that diet-related factors could independently influence mental symptoms. Therefore, to keep a healthy diet habits like reduction in fat intake might have beneficial effects on improvement mental health. In the future, we should focus on traditional mental health treatments as well as their diet and behavioral habits to improve the mental health of medical students. Education managers should also provide targeted interventions for improving mental support.

## Electronic supplementary material

Below is the link to the electronic supplementary material.

Supplementary Material 1

## Data Availability

The datasets used and/or analyzed in the present study are available from the corresponding author on reasonable request.

## Abbreviations

WHO: World Health Organization
SAS: Self-Rating Anxiety Scale
SDS: Self-Rating Depression Scale
KMO: Kaiser-Meyer-Olkin
COVID-19: Coronavirus Disease 2019
M ± SD: Mean ± Standard Deviation
IQR: Interquartile Range
MLR: Multiple Linear Regression
LBC: Left-Behind Children
GBD: Global Burden of Diseases.

## References

[1] GBD 2019 Mental Disorders Collaborators. Global, regional, and national burden of 12 mental disorders in 204 countries and territories, 1990–2019: a systematic analysis for the Global Burden of Disease Study 2019. Lancet Psychiatry. 2022 Feb;9(2):137–150. 10.1016/S2215-0366(21)00395-3 PMC8776563 35026139

[2] Smolen JR, Araújo EM (2017). Race/skin color and mental health disorders in Brazil: a systematic review of the literature. Cien Saude Colet.

[3] Bourbonnais R, Vézina M (1995). Mental health of white collar workers and the psychosocial environment at work. Sante Ment Que.

[4] Bertolote JM, Fleischmann A (2002). Suicide and psychiatric diagnosis: a worldwide perspective. World Psychiatry.

[5] Alzahrani M, Alfahaid F, Almansour M, Alghamdi T, Ansari T, Sami W, Otaibi TMA, Humayn AAA, Enezi MMA (2017). Prevalence of generalized anxiety disorder and major depression in health-care givers of disabled patients in Majmaah and Shaqra cities, Kingdom of Saudi Arabia. Int J Health Sci (Qassim).

[6] Chen L, Wang L, Qiu XH, Yang XX, Qiao ZX, Yang YJ, Liang Y (2013). Depression among Chinese university students: prevalence and socio-demographic correlates. PLoS ONE.

[7] Herting MM, Sowell ER (2017). Puberty and structural brain development in humans. Front Neuroendocrinol.

[8] Shao R, He P, Ling B, Tan L, Xu L, Hou Y, Kong L, Yang Y (2020). Prevalence of anxiety and depression and correlations between depression, anxiety, family functioning, social support and coping styles among Chinese medical students. BMC Psychol.

[9] Quek TT, Tam WW, Tran BX, Zhang M, Zhang Z, Ho CS, Ho RC (2019). The Global Prevalence of Anxiety Among Medical Students: A Meta-Analysis. Int J Environ Res Public Health.

[10] Puthran R, Zhang MW, Tam WW, Ho RC (2016). Prevalence of depression amongst medical students: a meta-analysis. Med Educ.

[11] Shuval K, Shachak A, Linn S, Brezis M, Reis S (2007). Evaluating primary care doctors’ evidence-based medicine skills in a busy clinical setting. J Eval Clin Pract.

[12] O’Hagan AD, Issartel J, Nevill A, Warrington G (2017). Flying Into Depression. Workplace Health Saf.

[13] Adhikari KPaudel,TBallav, Khanal P, Bhatta R, Paudel R, Bhusal S, Prem Basel (2022). Sleep quality and its correlates among undergraduate medical students in Nepal: A cross-sectional study. PLOS Glob Public Health February.

[14] Xie L, Luo H, Li M, Ge W, Xing B, Miao Q (2020). The immediate psychological effects of Coronavirus Disease 2019 on medical and non-medical students in China. Int J Public Health.

[15] Liu Z, Liu R, Zhang Y, Zhang R, Liang L, Wang Y, Wei Y, Zhu R, Wang F. Association between perceived stress and depression among medical students during the outbreak of COVID-19: The mediating role of insomnia. J Affect Disord. 2021 Sep 1;292:89–94. 10.1016/j.jad.2021.05.028 PMC8595067 34107425

[16] Halperin SJ, Henderson MN, Prenner S, Grauer JN (2021). Prevalence of Anxiety and Depression Among Medical Students During the Covid-19 Pandemic: A Cross-Sectional Study. J Med Educ Curric Dev.

[17] Muhammad Alfareed Zafar S, Junaid Tahir M, Malik M, Irfan Malik M, Kamal Akhtar F, Ghazala R (2020). Awareness, anxiety, and depression in healthcare professionals, medical students, and general population of Pakistan during COVID-19 Pandemic: A cross sectional online survey. Med J Islam Repub Iran.

[18] Liu Z, Qiao D, Xu Y, Zhao W, Yang Y, Wen D, Li X, Nie X, Dong Y, Tang S, Jiang Y, Wang Y, Zhao J, Xu Y (2021). The Efficacy of Computerized Cognitive Behavioral Therapy for Depressive and Anxiety Symptoms in Patients With COVID-19: Randomized Controlled Trial. J Med Internet Res.

[19] Zung WW, Richards CB, Short MJ (1965). Self-rating depression scale in an outpatient clinic. Further validation of the SDS. Arch Gen Psychiatry.

[20] Sun L, Sun LN, Sun YH, Yang LS, Wu HY, Zhang DD, Cao HY, Sun Y (2011). Correlations between psychological symptoms and social relationships among medical undergraduates in Anhui Province of China. Int J Psychiatry Med.

[21] Aniebue PN, Onyema GO (2008). Prevalence of depressive symptoms among nigerian medical undergraduates. Trop Dr.

[22] Mohajeri MH, La Fata G, Steinert RE, Weber P (2018). Relationship between the gut microbiome and brain function. Nutr Rev.

[23] Lof J, Smits K, Melotte V, Kuil LE (2022). The health effect of probiotics on high-fat diet-induced cognitive impairment, anxiety and depression: A cross-species systematic review. Neurosci Biobehav Rev.

[24] Bremner JD, Moazzami K, Wittbrodt MT, Nye JA, Lima BB, Gillespie CF, Rapaport MH, Pearce BD, Shah AJ, Vaccarino V. Diet. Stress and Mental Health. Nutrients. 2020 Aug 13;12(8):2428. 10.3390/nu12082428 PMC7468813 32823562

[25] Supe AN (1998). A study of stress in medical students at Seth G.S. Medical College. J Postgrad Med.

[26] Kang M, Choi SY, Jung M (2021). Dietary intake and nutritional status of Korean children and adolescents: a review of national survey data. Clin Exp Pediatr.

[27] Wang X, Liu A, Du M, Wu J, Wang W, Qian Y, Zheng H, Liu D, Nan X, Jia L, Song R, Liang D, Wang R, Wang P (2020). Diet quality is associated with reduced risk of hypertension among Inner Mongolia adults in northern China. Public Health Nutr.

[28] Chen W, Huang Y, Riad A (2021). Gender Differences in Depressive Traits among Rural and Urban Chinese Adolescent Students: Secondary Data Analysis of Nationwide Survey CFPS. Int J Environ Res Public Health.

[29] Liu H, Zhou Z, Fan X, Luo H, Wang D, Wang J, Shen C, Nawaz R (2021). A mixed method study to examine the mental health problems of college students who had left-behind experiences. J Affect Disord.

[30] Han L, Zhao SY, Pan XY, Liao CJ (2018). The impact of students with left-behind experiences on childhood: The relationship between negative life events and depression among college students in China. Int J Soc Psychiatry.

[31] Wang W, Dong Y, Liu X, Zhang L, Bai Y, Hagist S. The More Educated, the Healthier: Evidence from Rural China. Int J Environ Res Public Health. 2018 Dec 13;15(12):2848. 10.3390/ijerph15122848 PMC6313666 30551642

[32] Dekel S, Ein-Dor T, Berman Z, Barsoumian IS, Agarwal S, Pitman RK (2019). Delivery mode is associated with maternal mental health following childbirth. Arch Womens Ment Health.

[33] Lowe NK (2007). A review of factors associated with dystocia and cesarean section in nulliparous women. J Midwifery Womens Health.

[34] Dinis J, Bragança M (2018). Quality of Sleep and Depression in College Students: A Systematic Review. Sleep Sci.

[35] Löwenbrück F, Hess U (2021). Not all “caregivers” are created equal: Liking, caring and facial expression responses to the baby schema as a function of parenthood and testosterone. Biol Psychol.

