# Supplementary material for: Anxiety and depression and their interdependent influencing factors among medical students in Inner Mongolia: the cross-sectional survey
Source: BMC Med Educ. 2022 Nov 14;22:787. doi: 10.1186/s12909-022-03839-0 (PMC9662114; doi:10.1186/s12909-022-03839-0)
Supplement: Supplementary file 1 — Supplementary Material 1 [file 12909_2022_3839_MOESM1_ESM.docx]

**Note:** Please Mark “√” on the appropriate number according to your actual feeling in a week, do not miss any one item, also do not repeat the assessment on the same item; In addition, there are some reverse items in the scale, please pay attention to ensure the understanding of the score.

| **Items** | **None OR A little of the time** | **Some of the time** | **Good part of the time** | **Most OR All of the time** |
| --- | --- | --- | --- | --- |
| 1. I feel more nervous and anxious than usual | 1 | 2 | 3 | 4 |
| 1. I feel afraid for no reason at all | 1 | 2 | 3 | 4 |
| 1. I get upset easily or feel panicky | 1 | 2 | 3 | 4 |
| 1. I feel like I’m falling apart and going to pieces | 1 | 2 | 3 | 4 |
| 1. I feel that everything is all right and nothing bad will happen | 4 | 3 | 2 | 1 |
| 1. My arms and legs shake and tremble | 1 | 2 | 3 | 4 |
| 1. I am bothered by headaches, neck and back pains | 1 | 2 | 3 | 4 |
| 1. I feel weak and get tired easily | 1 | 2 | 3 | 4 |
| 1. I feel calm and can sit still easily | 4 | 3 | 2 | 1 |
| 1. I can feel my heart beating fast | 1 | 2 | 3 | 4 |
| 1. I am bothered by dizzy spells | 1 | 2 | 3 | 4 |
| 1. I have fainting spells or feel like it | 1 | 2 | 3 | 4 |
| 1. I can breathe in and out easily | 4 | 3 | 2 | 1 |
| 1. I get feelings of numbness and tingling in my fingers, toes | 1 | 2 | 3 | 4 |
| 1. I am bothered by stomachaches or indigestion | 1 | 2 | 3 | 4 |
| 1. I have to empty my bladder often | 1 | 2 | 3 | 4 |
| 1. My hands are usually dry and warm | 4 | 3 | 2 | 1 |
| 1. My face gets hot and blushes | 1 | 2 | 3 | 4 |
| 1. I fall asleep easily and get a good night’s rest | 4 | 3 | 2 | 1 |
| 1. I have nightmares | 1 | 2 | 3 | 4 |
| **Total Score** |  | | | |

Self-rating Anxiety Scale (SAS)

| **Items** | **A Little of the Time** | **Some of the time** | **Good part of the time** | **Most of the time** |
| --- | --- | --- | --- | --- |
| 1. I feel down-hearted and blue | 1 | 2 | 3 | 4 |
| 2. Morning is when I feel the best | 4 | 3 | 2 | 1 |
| 3.I have crying spells or feel like it | 1 | 2 | 3 | 4 |
| 4.I have trouble sleeping at night | 1 | 2 | 3 | 4 |
| 5.I eat as much as I used to | 4 | 3 | 2 | 1 |
| 6.still enjoy sex | 4 | 3 | 2 | 1 |
| 7.I notice that I am losing weight | 1 | 2 | 3 | 4 |
| 8.I have trouble with constipation | 1 | 2 | 3 | 4 |
| 9.My heart beats faster than usual | 1 | 2 | 3 | 4 |
| 10.I get tired for no reason | 1 | 2 | 3 | 4 |
| 11.My mind is as clear as it used to be | 4 | 3 | 2 | 1 |
| 12.I find it easy to do the things I used to | 4 | 3 | 2 | 1 |
| 13.I am restless and can't keep still | 1 | 2 | 3 | 4 |
| 14.I feel hopeful about the future | 4 | 3 | 2 | 1 |
| 15.I am more irritable than usual | 1 | 2 | 3 | 4 |
| 16.I find it easy to make decisions | 4 | 3 | 2 | 1 |
| 17.I feel that I am useful and needed | 4 | 3 | 2 | 1 |
| 18.My life is pretty full | 4 | 3 | 2 | 1 |
| 19.I feel that others would be better off if I were dead | 1 | 2 | 3 | 4 |
| 20.I still enjoy the things I used to do | 4 | 3 | 2 | 1 |
| **Total Score** |  | | | |

Self-Rating Depression Scale (SDS)
